# Does a short luteal phase correlate with an increased risk of miscarriage? A cohort study

**DOI:** 10.1186/s12884-022-05195-9

**Published:** 2022-12-09

**Authors:** Marguerite Duane, Karen Schliep, Christina A. Porucznik, Shahpar Najmabadi, Joseph B. Stanford

**Affiliations:** 1 Department of Family Medicine, Georgetown University, Washington, DC USA; 2 Executive Director, Fertility Appreciation Collaborative to Teach the Science (FACTS), Lancaster, PA USA; 3 Office of Cooperative Reproductive Health, Division of Public Health, Department of Family and Preventive Medicine, University of Utah, Salt Lake City, UT USA

**Keywords:** Miscarriage, Early pregnancy loss, Short luteal phase, Progesterone, Pregnancy, Maternal age, Reproductive life plan, Fertility

## Abstract

**Background:**

Miscarriage is defined as spontaneous loss of pregnancy prior to 20 weeks gestation. With an estimated risk of 15% of clinically confirmed pregnancies ending in miscarriage, it is the most common adverse event in pregnancy. Woman’s age is the primary risk factor for miscarriage, while medical conditions, including hormonal abnormalities, are also associated. Progesterone is essential for maintaining pregnancy. A short luteal phase may reflect inadequate levels of progesterone production, but it is unclear whether a short luteal phase correlates with an increase in the risk of miscarriage.

**Methods:**

Using a cohort study design, we conducted a secondary data analysis from four cohorts of couples who used a standardized protocol to track biomarkers of the female cycles. A short luteal phase was defined as less than 10 days, with < 11, < 9, and < 8 days as alternate definitions in sensitivity analyses. We included women who experienced a pregnancy with a known outcome, identified the length of the luteal phase in up to 3 cycles prior to conception and assessed the relationship with miscarriage using a modified Poisson regression analysis, adjusting for demographic characteristics, smoking, alcohol use and previous pregnancy history.

**Results:**

In our sample of 252 women; the overall miscarriage rate was 18.7%. The adjusted incident risk ratio of miscarriage in women who had at least one short luteal phase < 10 days, compared to those who had none, was 1.01 (95% CI: 0.57, 1.80) Similar null risk was found when assessing alternative lengths of short luteal phase. Women who had short luteal phases < 10 days in all 3 cycles prior to the conception cycle had an incident risk ratio of 2.14 (95% CI: 0.7, 6.55).

**Conclusions:**

Our study found that a short luteal phase in the three cycles prior to conception was not associated with higher rates of miscarriage in an international cohort of women tracking their cycles, but our sample size was limited. Further research to determine if short luteal phases or luteal phase deficiency is associated with early pregnancy losses among preconception cohorts with daily tracking of cycle parameters, in addition to progesterone and human chorionic gonadotropin levels, is warranted. Additionally, future studies should include women with recurrent short luteal phases as a more likely risk factor than isolated short luteal phases.

## Plain English summary

This study looks at whether women have a higher risk of miscarriage if the second half of their menstrual or reproductive cycle is shorter than normal. The second half of the cycle, referred to as the luteal phase, is normally 11 to 16 days long. If the luteal phase is too short this may suggest a woman does not have enough progesterone, the hormone essential for maintaining pregnancy.

This study included 252 pregnant women for whom we had data on the length of at least one luteal phase prior to pregnancy and for whom we knew the outcome of the pregnancy. Almost one-fifth of the women in our study had a miscarriage. However, we found there was no difference in the risk for miscarriage if women had a short or a normal luteal phase.

## Introduction

Miscarriage, spontaneous pregnancy loss < 20 weeks gestational age, is the most common adverse event in early pregnancy, occurring in 10–20% of clinically recognized pregnancies [1–3] and up to 30% of all pregnancies including unrecognized pregnancies [4–6]. Since miscarriage in recognized pregnancies is associated with significant physical and psychological morbidity, including post-traumatic stress disorder, anxiety and depression [3, 7, 8], it is important to identify women who may be at increased risk for miscarriage, especially recurrent miscarriage, currently defined as ≥ 2 pregnancy losses < 20 weeks gestational age [3, 9, 10].

The majority of all miscarriages is due to chromosomal abnormalities in the embryo [3, 11]. A number of medical conditions are also associated with sporadic and recurrent miscarriage, including uterine malformations, maternal thrombophilia, and immune dysfunction. Endocrine disorders, including insufficient luteal progesterone production, have also been suggested to be associated with early pregnancy losses. Specifically, insufficient levels or duration of progesterone production may result in a luteal phase deficiency, which inhibits maintenance of a normal secretory endometrium allowing for normal embryo implantation and growth [12]. A recent Cochrane review suggests that there may be a reduction in the number of miscarriages for women with risk factors for miscarriage (including prior miscarriage or bleeding in early pregnancy), who are given progestogen supplementation compared to placebo [13]. While inadequate progesterone levels are associated with a short luteal phase [14], to date there have been few studies investigating the link between the length of the luteal phase and risk of miscarriage [3, 7].

The aim of this study is to address this data gap by establishing whether a short luteal phase (defined as < 10 days after estimated day of ovulation through the end of the cycle) correlates with an increased risk of miscarriage in women who are tracking their menstrual cycles [14]. We hypothesize that a short luteal phase increases the risk of miscarriage, after taking into account important confounding factors. Furthermore, we hypothesize miscarriage risk will be augmented by increased number of cycles with short luteal phases prior to conception.

## Methods

### Study population and design

This study was a secondary data analysis from four cohorts of sexually active women, and their male partners, who were tracking their cycles using the Creighton Model Fertility Care System (CrM). The CrM teaches women and couples to observe and interpret patterns of cervical fluid secretion and vaginal bleeding to identify the timing of ovulation and the fertile window. The cohorts include 1) the Creighton Model Effectiveness, Intentions and Behaviors Assessment (CEIBA) (2009–2013), a prospective cohort of 293 women, without known subfertility, designed to evaluate pregnancy effectiveness rates and intentions with use of the CrM to avoid pregnancy or to conceive [15]; 2) Creighton Model MultiCenter Fecundability Study (CMFS) (1990–1996), a retrospective cohort of 309 women using CrM designed to determine the relationship between vulvar mucus observations and the day and cycle specific probabilities of conception (some with normal fertility and some with subfertility) [16]; 3) the international NaProTechnology Evaluation and Surveillance of Treatment (iNEST) study (2006–2016), a longitudinal cohort study of 834 couples with subfertility seeking treatment [17]; and 4) the Time to Pregnancy in Normal Fertility (TTP) (2003–2006), a parallel randomized trial of 68 couples with proven fertility, aimed to determine the impact of the use of CrM on time to conceive [18]. All studies received IRB approval through the University of Utah, and written informed consent was obtained for the prospective studies (CEIBA, iNEST, and TTP).

All four cohort studies included women, and their male partners, between the ages of 18 and 40 years old, except iNEST, which did not have an upper age limit, and TTP, which had an upper limit of 35 years [15]. The CMFS study recruited couples with both normal fertility and subfertility, whereas, the iNEST study only recruited couples with subfertility or a history of miscarriage. The CEIBA and TTP studies aimed to include couples of normal fecundity, so women could not be taking any hormonal medications or drugs that could affect fertility, and both partners had to be free of any diagnosis that could potentially cause subfertility. Additional entry criteria included regular menstrual cycles, including having had at least one menstrual bleed (CEIBA) or two menstrual bleeds (TTP) since discontinuing oral contraceptives or if post-partum (CEIBA). The combined total number from these cohorts is 1504 women. Since our study aimed to assess the impact of a short luteal phase on the risk of miscarriage, we included women who became pregnant for whom we had a known pregnancy outcome and at least one luteal phase length documented in one of the three cycles prior to the conception cycle. We examined miscarriage rates for each individual cohort, as well as for the overall study population. The total number of pregnant women from all 4 cohorts was 365 (CEIBA—106, TTP—53, CMFS—146 and iNEST—60). The pregnancy outcome was known for 288 women (CEIBA—84, TTP—53, CMFS—91 and iNEST – 60) and we had data on the length of at least one luteal phase prior to conception for 252 women (CEIBA—79, TTP—42, CMFS—85 and iNEST—46).

### Exposure and outcome variables

Via the daily CrM cycle charts, we identified all cycles of conception and then analyzed the cycles immediately preceding, up to 3, to determine the length of the luteal phase in each cycle and the number of cycles with short luteal phases. Our primary analyses defined a short luteal phase as 10 days or less after peak (estimated day of ovulation) through the last day of the cycle. The primary outcome variable is miscarriage by self-report, defined as the spontaneous loss of a recognized pregnancy prior to 20-weeks gestation.

### Potential confounding variables

We identified potential confounding variables a priori via a literature review, which included woman’s age, race/ethnicity, work history, prior pregnancy history (nulligravida versus history of live births versus miscarriages), smoking and alcohol use. All of these variables were assessed by self-report of the women at baseline. Since low production of progesterone (level and/or duration) may cause a short luteal phase, exogenous administration of progesterone or human chorionic gonadotropin (HCG) may also be a confounding variable since it can mitigate the impact of low progesterone production. Where the data were available, we assessed if the use of supplemental progesterone or HCG during the cycles prior to conception or during the pregnancy itself is associated with a decreased risk of miscarriage.

### Data analysis

We report characteristics of our population including socio-demographics, pregnancy history and lifestyle factors by whether women had a miscarriage during follow-up (Table 1). For our primary aim, we used modified Poisson regression analysis models to generate risk ratios (RR) and 95% confidence intervals (CI) to determine if presence or absence of short luteal phase was associated with risk of miscarriage. We report unadjusted and adjusted models. Final models adjusted for age and we also stratified based on previous pregnancy history. Effect modification by age was assessed through stratified results and the Wald test.

**Table 1:** Demographic and reproductive characteristics by miscarriage rates

	**Miscarriage** ***N* = 48 (19%)**	**No Miscarriage** ***N* = 204 (81%)**	**Total** ***N* = 252**	***P*-value** **(chi-square)**
**Study Source—Data Set**				0.008
CEIBA	13 (16.5%)	66 (83.5%)	79	
TTP	6 (14.3%)	36 (85.7%)	42	
CMFS	12 (14.1%)	73 (85.9%)	85	
iNEST	17 (37%)	29 (63%)	46	
**Maternal age (y)**				0.003
< 30	23 (13.8%)	144 (86.2%)	167	
≥ 30	25 (29.4%)	60 (70.6%)	85	
Missing			0	
**Paternal age (y)**				0.006
< 30	17 (12.7%)	117 (87.3%)	134	
≥ 30	30 (26.3%)	84 (73.7%)	114	
Missing	1	3	4	
**Race & ethnicity**				0.819
White (non-Hispanic)	42 (18.6%)	184 (81.4%)	226	
Hispanic	1 (14.3%)	6 (85.7%)	7	
Other	1 (11.1%)	8 (88.9%)	9	
Missing	4	6	10	
**Federal poverty level, adjusted by year**				0.587
< 150%	3 (23.1%)	10 (76.9%)	13	
150%-200%	2 (11.1%)	16 (88.9%)	18	
≥ 200%	22 (13.4%)	142 (86.6%)	164	
Missing	21	36	57	
**Completed education**				0.534
High / Vocational / Technical school graduate or less	4 (25%)	12 (75%)	16	
Some college	6 (13.3%)	39 (86.7%)	45	
College graduate	34 (18.8%)	147 (81.2%)	181	
Missing	4	6	10	
**Occupational status**				0.692
Professional	18 (17.6%)	84 (82.4%)	102	
Technical / Skilled or unskilled laborer	2 (11.1%)	16 (88.9%)	18	
Clerical / Sales	6 (23.1%)	20 (76.9%)	26	
Homemaker	4 (10%)	36 (92.3%)	40	
Student	4 (22.2%)	14 (73.7%)	18	
Other	3 (16.7%)	15 (83.3%)	18	
Missing	11	19	30	
**Employed**				0.336
Yes	26 (16.4%)	133 (83.7%)	159	
No	5 (10.6%)	42 (89.4%)	47	
Missing	17	29	46	
**Smoking**				0.537
Yes	2 (22.2%)	7 (77.8%)	9	
No	16 (14.5%)	94 (85.5%)	110	
Missing	30	103	133	
**Drinking**				0.511
Yes	12 (16.9%)	59 (83.1%)	71	
No	6 (12.5%)	42 (87.5%)	48	
Missing	30	103	133	
**Age at first pregnancy (y)**				0.740
Never pregnant	19 (16.7%)	95 (83.3%)	114	
≤ 19	1 (5%)	19 (95%)	20	
20–24	3 (18.8%)	13 (81.3%)	16	
25–29	2 (15.4%)	11 (84.6%)	13	
≥ 30	6 (14.3%)	36 (85.7%)	42	
Missing data:	17	30	47	
**Parity**				0.470
No previous pregnancy	19 (16.7%)	95 (83.3%)	114	
Previous pregnancy	12 (13.0%)	80 (87%)	92	
Missing data	17	29	46	
**Previous live birth**				0.489
None	20 (16%)	105 (84%)	125	
At least one	10 (12.5%)	70 (87.5%)	80	
Missing data:	18	29	47	
**Previous spontaneous abortion**				0.515
None	23 (13.9%)	143 (86.1%)	166	
At least one	7 (18%)	32 (82%)	39	
Missing data	18	29	47	
**Medication in luteal phase (progesterone/HCG)**				
No Medication	44 (19.3%)	184 (80.7%)	228	
Medication	4 (16.7%)	20 (83.3%)	24	

### Sensitivity analysis

We performed a sensitivity analysis with alternate definitions of short luteal phase lengths (<11, 9 and 8, and 7 days, respectively), in relation to miscarriage for our modified Poisson regression analyses. This was designed to assess whether a more stringent definition of luteal phase defect has a stronger effect size for either outcome.

## Results

As noted in Table 1, we had a total of 252 participants, with 167 women < 30 and 85 women > 30 years. The overwhelming majority of women were white (89.7%), college educated (71.8%), and lived well above (> 200%) the federal poverty level (65.1%). The overall miscarriage rate in our study was 18.7%. Table 1 illustrates miscarriage rates based on the demographic characteristics, including study cohorts, and reproductive history of our study population. Women in the iNEST cohort (couples with subfertility) had a significantly higher rate of miscarriage at 37.0%. Our study confirmed that maternal age is associated with pregnancy loss as the rates of miscarriage were almost double in women over 30 (29.4%) as compared to women under 30 (13.8%, p-value 0.03). Similarly, paternal age was also shown to be a significant risk factor with a miscarriage rate of 26.3% for women whose male partners were > 30 years vs 12.7% for male partners < 30 years (*p*-value 0.006). However, since paternal age is highly correlated with maternal age (Pearson *r* = 0.55, *p* < 0.001), it is likely acting as a proxy for maternal age in univariate analysis. The rate of miscarriage differed by various demographic characteristics, but none of the differences were statistically significant, outside of age (Table 1).

With regards to a woman’s reproductive history, women who had previously been pregnant or had a live birth had slightly lower rates of miscarriage, and women with the history of previous miscarriage had slightly higher rates of miscarriage, although not statistically significant (see Table 1). We examined the impact of treatment with progesterone and/or human chorionic gonadotropin hormones in any of the 3 cycles prior to conception and found that women who received these medications had a slightly lower rate of miscarriage (16.7% vs 19.3%) as seen in Table 1, also not statistically significant. However, the total number of women who reported receiving these medications was small.

Table 2 illustrates the results of our adjusted modified Poisson regression analysis which found no difference in risk of miscarriage among women with luteal phase lengths < 8 days (IRR = 1.17, 95% CI: 0.57, 2.38), < 9 days (IRR = 1.03, 95% CI:), < 10 days (IRR = , 95% CI:) or < 11 days (IRR = , 95% CI:). Based on the Wald test, there was no significant interaction between women’s age and short luteal phase (*p* = 0.42).

**Table 2:** Incident risk ratio of miscarriage in the presence or absence of short luteal phases or anovulatory cycles in any of the prior 3 cycles, adjusted for age

Luteal phase	Women	Miscarriages	Percent	IRR	95% CI	
> = 11 days (referent)	138	28	20.3%			
< 11 days	113	20	17.7%	0.87	0.52	1.46
< 10 days	111	20	18.0%	1.01	0.57	1.8
< 9 days	56	11	19.6%	1.03	0.57	1.9
< 8 days	32	7	21.9%	1.17	0.57	2.38
Anovulatory	69	14	20.3%	1.01	0.57	1.8

Table 3 illustrates the risk of miscarriage based on the total number of short luteal phases < 10 days prior to pregnancy, in up to 3 cycles. There was an incident risk ratio of miscarriage of 2.14 (95%CI: 0.70, 6.55) if a woman has luteal phases less than 10 days in all 3 cycles prior to pregnancy; however, this was based on only 5 women and 2 miscarriages.

**Table 3:** Incident risk ratio of miscarriage based on the number of short luteal phases in any of the 3 cycles prior to conception

**# of short luteal phases < 10 days**	**Women**	**Miscarriages**	**Percent**	**IRR**	**95% CI**	
0	171	32	18.7%	1		
1	59	13	22.0%	1.18	0.66	2.09
2	16	1	6.3%	0.33	0.05	2.29
3	5	2	40.0%	2.14	0.7	6.55

Table 4 indicates that the risk of miscarriage in women age 30 years and older is independent of the length of the luteal phase. Specifically, the risk of miscarriage is more than twice as high in women 30 and older even if they had no short luteal phases versus at least one short luteal phase less than 11 days, 10 days, 9 days or 8 days.

**Table 4:** Risk of Miscarriage in women > age 30 stratified by previous luteal phase length in the 3 cycles prior to conception

Any short luteal phase	Percent of women > 30 with miscarriage	IRR –Women > 30^a^	95% CI	
None	34.5%	2.13	1.28	3.50
< 11 days	31.5%	2.12	1.29	3.52
< 10 days	30.3%	2.09	1.27	3.46
< 9 days	25.3%	2.13	1.29	3.54
< 8 days	28%	2.12	1.28	3.49

^a^Reference group for the IRR is women <  = 30 years

Table 5 illustrates the risk of miscarriage based on luteal phase length and previous pregnancy or miscarriage history. Women with a previous pregnancy or miscarriage did have higher rates of miscarriage if they had a short luteal phase. In contrast, women with no previous pregnancy or miscarriage had lower rates of miscarriage if they had a short luteal phase.

**Table 5:** Miscarriage rates in women with normal or short luteal phases (< 10 days) based on previous pregnancy or miscarriage history

	**No previous pregnancy**	**Previous pregnancy**	**No previous miscarriage**	**Previous miscarriage**
Normal Luteal Phase	12/63 (19.0%)	5/48 (10.4%)	14/91 (15.4%)	2/19 (10.5%)
Short Luteal Phase	7/51(13.7%)	7/44 (15.9%)	9/75 (12.0%)	5/20 (25%)

## Discussion

The goal of this study was to assess if there is an association between luteal phase length, a potentially modifiable risk factor, and miscarriage rates. Since the length of the luteal phase is determined by the levels and duration of progesterone, the primary hormone responsible for preparing and maintaining the uterine lining in the second half of the cycle, we hypothesized that women with short luteal phases would have a higher risk of miscarriage. However, our results showed no difference in miscarriage rates with at least one short luteal phase in the three cycles prior to the conception cycle. We also found no impact of prior anovulatory cycles. We did note a possible increased relative risk of miscarriage if women had a short luteal phase in all 3 tracked cycles preceding conception, but this was based on only 5 women and 2 miscarriages in that group. The overall miscarriage rate in our study was 18.7%, which is consistent with the pooled risk of miscarriage of 15.3% in all recognized pregnancies, from a review of nine large cohort studies in Europe and North America [3]. However, this overall rate of 18.7% may not reflect rates in sub-populations of patients with subfertility or history of miscarriage.

It’s well established that women or couples with a history of subfertility have a higher risk of miscarriage, particularly recurrent miscarriage, and this is consistent with the higher rate of miscarriage in the iNEST group, since the iNEST study enrolled only couples with subfertility [17]. One possible bias could arise if women with subfertility take longer to conceive, and therefore are more likely to have 3 tracked cycles prior to conception, while also possibly being at higher risk of miscarriage. One study has shown that pregnancy rates were decreased in the first 6 months after an isolated short luteal phase [9].

Currently, the American Society for Reproductive Medicine and the American College of Obstetricians and Gynecologists do not recommend workup or management of miscarriage until after the second consecutive clinical early pregnancy loss [19, 20]. Yet, research shows that supplementation with progesterone is associated with increasing live birth rates in some women with bleeding in early pregnancy, and/or who have had previous miscarriages [13, 21]. Causes of recurrent miscarriage are known to be different than causes of a single miscarriage [3]. Our results may suggest that women who have had prior miscarriages may be more likely to have a hormonal imbalance, such as insufficient progesterone, that could also be associated with a shorter luteal phase. Although the number of women who were known to receive hormonal support in our study was relatively small and may have been underreported, there was an absolute lower rate of miscarriage of about 2.7% among those women who received progesterone (or HCG). Because miscarriage is common and because a short luteal phase may reflect inadequate levels of progesterone, identifying an association between luteal phase length and the risk of miscarriage would have high relevance to clinical practice and public health. Although controversy exists about whether treatment of luteal phase deficiency improves outcomes, the absolute lower rate of miscarriage in women who received hormonal support in our study is consistent with prior clinical trials which have suggested that women with history of prior miscarriage and/or bleeding in early pregnancy are more likely to benefit from progesterone treatment [12, 21].

Since the incidence of short luteal phases and miscarriages are both highest at the ends of the reproductive age spectrum, we evaluated whether the miscarriage risk associated with age was related to luteal phase length [22]. However, our results found no impact of luteal phase length on miscarriage rates in women over age 30. This highlights that there may be a multitude of factors that influence the risk of miscarriage, and that impact of age on miscarriage risk is probably through mechanisms other than progesterone in the luteal phase.

Consistent with previous research, our study confirmed that increased maternal age is associated with miscarriage, as is increased paternal age, but since these two were highly correlated, paternal age may simply be a proxy for maternal age in our study [11, 23]. Typically, age is not considered a modifiable risk factor, since one cannot turn back the clock. However, it may be important to educate women and men about the association between age and miscarriage as part of the reproductive life planning process [24]. A reproductive life plan encourages women and men to consider their preferences about whether and when to have children and should also take into consideration the desired number and spacing of children, maternal health and family health histories, as well as maternal and paternal ages [24, 25]. Although increased maternal age is noted as a risk factor for subfertility, the increasing rate of miscarriage with age is rarely mentioned. This lack of information may lead to regret about childbearing decisions for both women and men, as one study showed [26]. Therefore, efforts should be made to increase fertility knowledge, including the importance of age as a risk factor for miscarriage. Additionally, teaching women to chart their cycle with a fertility awareness-based method may also serve to increase their knowledge of their fertility and overall reproductive health [27].

Our study sought to address a very common adverse medical event that is rarely studied with the goal of identifying a novel risk factor that can be identified with fertility charting methods and may be modifiable. Additional strengths of our study included our ability to access four unique datasets representing a population of patients from the US and Europe over a 20-year period. Limitations of our study included a smaller than expected sample size that may be underpowered to detect differences among women who experienced a miscarriage as compared to those that did not. Our study was limited to largely white, non-Hispanic women. Finally, we did not have the gestational age at the time of miscarriage for the majority of women. However, statistically the highest rate of miscarriage is highest in the first trimester (> 80%) and we hypothesized that this is where a short luteal phase may have the greatest impact. Future research among more ethnically and racially diverse populations is needed. We had a limited number of cycles preceding the pregnancy cycle to adequately assess the impact of the number of short luteal phases on the outcome of the pregnancy, especially in women with two short luteal phases. Additionally, although women were generally advised to do a pregnancy test if their luteal phase was 16 days or more, if there was a loss prior to that time or women did not do a pregnancy test, it is possible that some early miscarriages were not captured. A larger sample size may have strengthened the study findings. Finally, we did not have information about other potential risk factors or causes for miscarriage, such as autoimmune or endocrine disorders that may have affected miscarriage rates.

## Conclusion

Since miscarriage is the most common adverse event in early pregnancy and it is associated with significant physical and psychological morbidity, it is important to identify women who may be at increased risk. This study was designed to assess whether a short luteal phase as reflected in cycle charting is correlated with an increased risk of miscarriage, but the results did not support an association. Further research with larger sample sizes is required to determine if recurrent short luteal phase (or other indicator of luteal phase deficiency, such as recurrent premenstrual spotting) is associated with early pregnancy losses before 20 weeks among preconception cohorts with daily tracking of menstrual cycle parameters, in addition to progesterone and human chorionic gonadotropin levels.

## Data Availability

Data used for this analysis is available from the corresponding author on reasonable request.

## Abbreviations

CrM: Creighton Model Fertility Care System (CrM)
CEIBA: Creighton Model Effectiveness, Intentions and Behaviors Assessment
CMFS: Creighton Model MultiCenter Fecundability Study
iNEST: International NaProTechnology Evaluation and Surveillance of Treatment
TTP: Time to Pregnancy in Normal Fertility
